# Establishment and Characterization of an Immortalized Porcine Oral Mucosal Epithelial Cell Line as a Cytopathogenic Model for Porcine Circovirus 2 Infection

**DOI:** 10.3389/fcimb.2019.00171

**Published:** 2019-05-21

**Authors:** Hongjie Cui, Wulong Liang, Dahui Wang, Kangkang Guo, Yanming Zhang

**Affiliations:** ^1^College of Veterinary Medicine, Northwest A&F University, Yangling, China; ^2^School of Life Science, Shanxi University, Taiyuan, China; ^3^School of Agriculture and Forestry Engineering, Tongren University, Tongren, China

**Keywords:** immortalization, cell model, porcine oral mucosal epithelial cell, porcine circovirus 2, cytopathic effect

## Abstract

Porcine circovirus 2 (PCV2) is a major etiological agent for porcine circovirus-associated diseases and causes enormous economic losses in domestic and overseas swine production. However, there are currently no suitable cell models to study the cytopathic effects (CPE) of PCV2 *in vitro*, which severely restricts the study of PCV2 pathogenesis. In the present study, we established an immortalized porcine oral mucosal epithelial cell line (hTERT-POMEC) by introducing the hTERT gene into primary porcine oral mucosal epithelial cells (POMECs) derived from a neonatal, unsuckled piglet. The hTERT-POMEC cells have a homogeneous cobblestone-like morphology and retain the basic physiological properties of primary POMECs. No chromosome abnormality and tumorigenicity transformation was observed in immortalized hTERT-POMECs. Viral infection assays demonstrated that PCV2 propagated and caused CPE in hTERT-POMECs. We conclude that the immortalized cell line hTERT-POMEC is a crucial tool for further research into the pathogenesis of PCV2.

## Introduction

Porcine circovirus 2 (PCV2), a member of the genus *Circovirus* within the *Circoviridae* family (Mankertz et al., 1997; Lv et al., 2014; Wen, 2016), is the primary causative agent of porcine circovirus-associated diseases (PCVADs) in swine (Darwich et al., 2004; Chae, 2005; Opriessnig et al., 2007). PCV2 is widely distributed throughout the world and causes enormous economic losses in swine production (O'Dea et al., 2011; Novosel et al., 2012; Wilfred et al., 2018). Thus, characterization of PCV2 pathogenesis is necessary. Currently, PK-15 cells are the most frequently used model to study PCV2 in the laboratory (Zhu et al., 2007; Dvorak et al., 2013; Gan et al., 2016; Huang et al., 2018). However, PK-15 cells are not sufficiently permissive to PCV2 infection and show no CPE with low viral titers (Zhu et al., 2007). Therefore, this lack of a productive cell model is a major restriction to further study of the pathogenesis of PCV2.

Porcine oral mucosal epithelial cells (POMECs) are located in the outermost layer of the oral and nasal cavity, and are one of the first types of cells that encounter PCV2 during natural infection. Originally, it was thought that OMECs serve only as a physical barrier against invading pathogens (Squier and Kremer, 2001). However, recently it has become increasingly apparent that OMECs are capable of triggering an immune response similar to cells of the myeloid lineage (Presland and Jurevic, 2002; Feller et al., 2014; Bierbaumer et al., 2018), thus playing a crucial role in the active recognition of microbes. Accordingly, the oral epithelium is able to secrete a variety of defense effector molecules (Diamond et al., 2008) and to orchestrate an immune inflammatory response to activate myeloid cells in the submucosal layers to clear the invading pathogens (Cutler and Jotwani, 2006; Abusleme and Moutsopoulos, 2017; Nassar et al., 2018). Isolation and cultivation of primary POMECs are cumbersome procedures that are time-consuming and costly. Additionally, normal POMECs usually fall into senescence after 12 generations of culture *in vitro*. Creating a stable POMEC line is thus urgently needed for basic research.

Telomeres become progressively shorter as cells proliferate and may be the main cause of senescence (Harley, 1991; di Fagagna et al., 2003). Overexpression of human telomerase reverse transcriptase (hTERT) alone is sufficient to prevent telomere shortening and extends the lifespan of cells (Halvorsen et al., 1999; Hong et al., 2007). Several immortalized cell lines have been established by exogenous expression of *hTERT* gene (Hong et al., 2007; He et al., 2009; Dong et al., 2013; Zhang et al., 2016; An et al., 2017).

The objective of the present study was to establish an immortalized POMEC line and to test whether it could be a useful tool for the study of PCV2 pathogenesis. pCI-neo-hTERT plasmids were successfully introduced into primary POMECs, and prolonged the lifespan of POMECs. The immortalized hTERT-POMECs retained critical morphologic and key physiological characteristics of primary POMECs, and were able to divide and proliferate without chromosome abnormality or tumorigenic transformation. Viral infection assays indicated that hTERT-POMEC may serve as a suitable tool to explore the pathogenesis of PCV2.

## Results

### Immortalized POMECs Maintained the Morphological Features of Primary Cells

**Figure 1:**
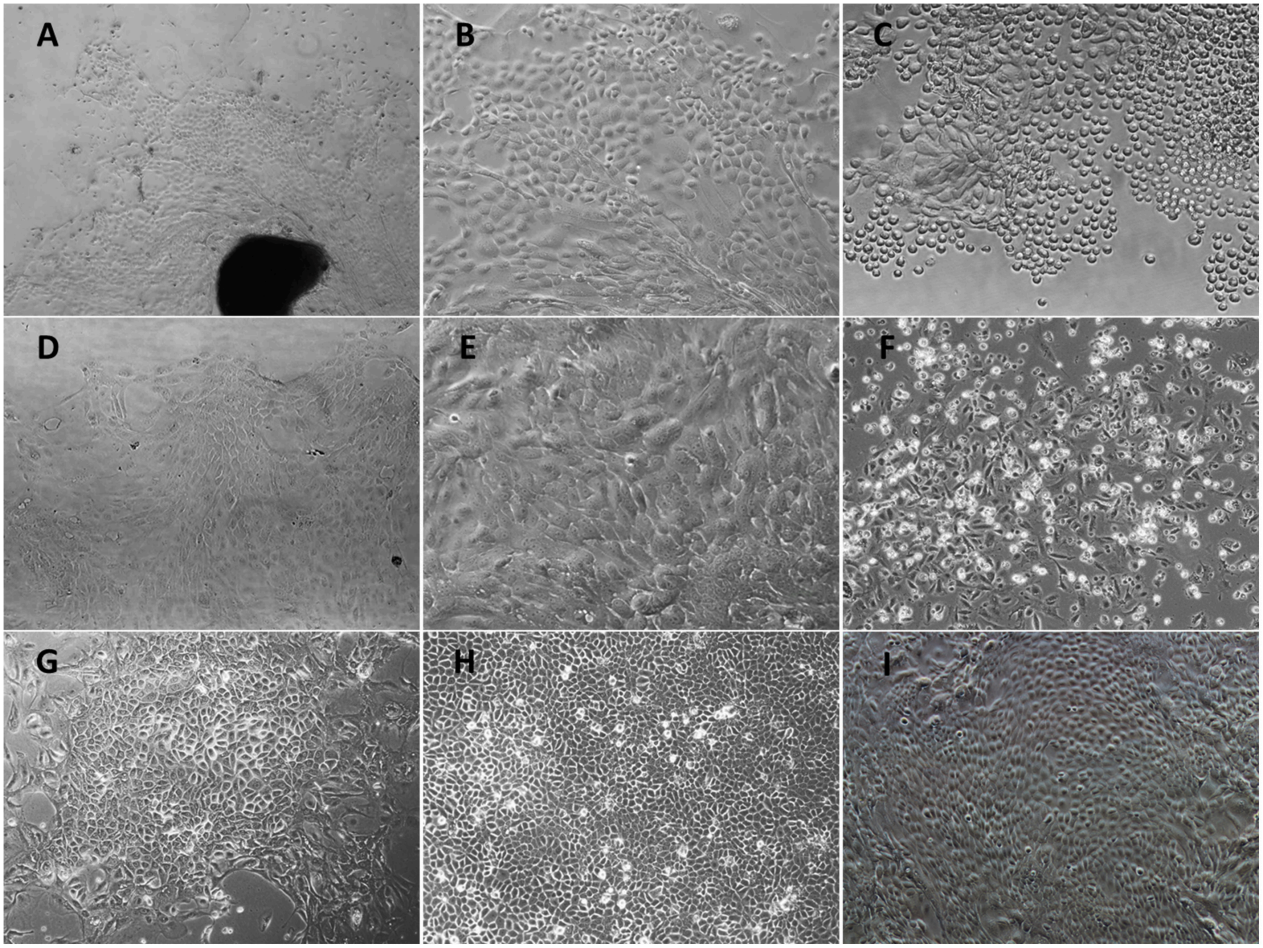
Morphological features of primary POMECs and hTERT-POMECs. **(A)** Primary tissue culture of POMECs at day 5 (×100 magnification). The black and dark area represents the epidermal tissue where POMECs derive from. **(B)** Morphology of primary POMECs (×200 magnification). **(C)** Subcultivation of primary POMECs by trypsin digestion (×200 magnification). **(D)** Morphology of third passage POMECs (×100 magnification). **(E)** Morphology of ninth passage POMECs (×200 magnification). **(F)** Senescent death of 12th passage POMECs (×100 magnification). **(G)** G418-resistant cell clone (×100 magnification). **(H,I)** Morphology of 35th and 110th passage hTERT-POMECs (×100 magnification).

After treatment with 0.25% dispase II solution, the separated upper epithelial tissues without fibroblasts were cut into small fragments and plated in collagen-coated culture flasks. Following 5 days incubation, several cellular aggregates formed circular proliferating foci (Figure 1A) and expanded into a confluent cell layer within 12 days. Most cells were monolayer except for occasional stratified layers. The primary POMECs exhibited a homogeneous cobblestone-like morphology (Figure 1B). Few fibroblasts were scraped out using a cell scraper. After digestion with trypsin, cells became round (Figure 1C) and were subcultured at a ratio of 1:3. Early passage (1–6) cells reached confluence within 4 days. However, an obvious reduction in propagation rate was observed after the ninth passage. The third and ninth passage POMECs still maintained the typical epithelial morphology without visible change (Figures 1D,E), while the 12th passage cells became senescent and rounded to death (Figure 1F).

To establish an immortalized POMEC line, the second or third passage POMECs were transfected with pCI-neo-hTERT plasmids, and selected with G418 for >15 days. When the control cells were all dead, a few transfected cells survived with drug resistance and formed colonies (Figure 1G). We referred to the immortalized cells as hTERT-POMECs. The 35th and 110th passaged hTERT-POMECs maintained the cobblestone-like morphology (Figures 1H,I). To date, the established hTERT-POMEC cell line has been cultured for >150 passages.

### hTERT-POMECs Maintained the Biological Features of Primary POMECs

**Figure 2:**
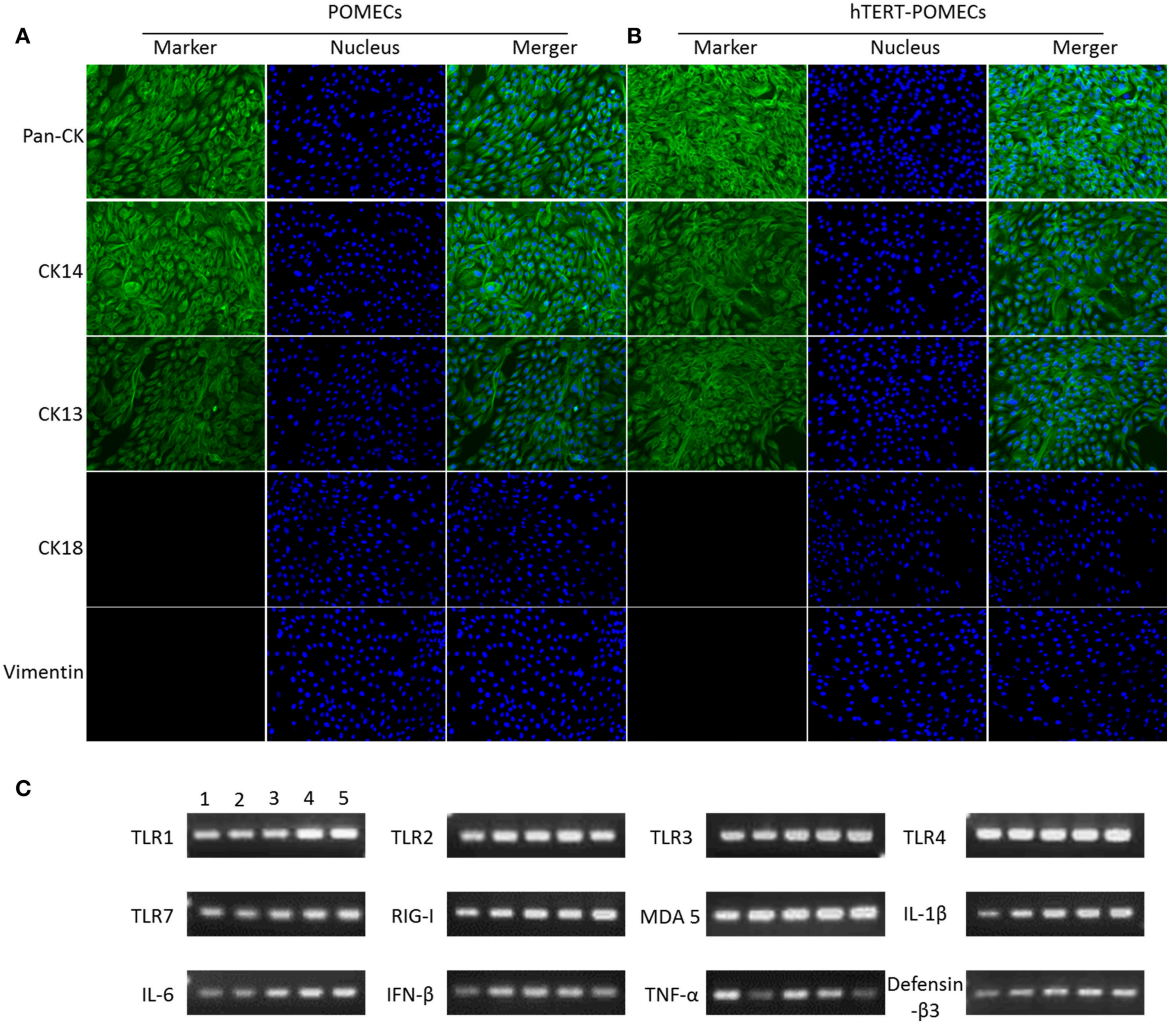
Identification of primary POMECs and hTERT-POMECs. **(A,B)** Immunofluorescence identification. Third passage POMECs and 80th passage hTERT-POMECs were stained with pan-cytokeratin, cytokeratin 14, cytokeratin 13, cytokeratin 18, or vimentin (×200 magnification). **(C)** Certain immune-related genes were detected qualitatively by RT-PCR in primary POMECs and hTERT-POMECs. Line1: third passage POMECs; Line 2–5: 30th, 50th, 80th, 100th passage hTERT-POMECs, respectively.

Keratin expression is the key feature of epithelial cells (Hosaka et al., 1985; Shabana et al., 1991; Bragulla and Homberger, 2009). Immunofluorescence assays were undertaken to examine the expression of cytokeratins in the primary POMECs and hTERT-POMECs. We detected the positive expression of pan-cytokeratin in both cells, which was an indicator of epithelial cell origin (Figures 2A,B). Cytokeratin 14 was detected (Figures 2A,B), which is usually found as a heterodimer with type II cytokeratin 5 and expressed in the basal proliferative layer of oral mucosal epithelial cells (Shabana et al., 1991; Belaldavar et al., 2016). Cytokeratin 13 was detected in both cells (Figures 2A,B), which paired with keratin 4 is generally expressed by differentiating (suprabasal) cells of non-cornified stratified epithelia (Shabana et al., 1991; Belaldavar et al., 2016). Cytokeratin 18 and vimentin, the major biomarkers expressed in simple epithelia and fibroblasts, respectively, were not detected in the primary POMECs and hTERT-POMECs (Figures 2A,B).

Some immune-related genes, such as TLR1, TLR2, TLR3, TLR4, TLR7, RIG-I, MDA 5, IL-1β, IL-6, IFN-β, TNF-α, and defensin-β3, were detected qualitatively by PCR in both the primary POMECs and hTERT-POMECs (Figure 2C). These results indicated that hTERT-POMECs maintained the biological features of primary POMECs.

### hTERT-POMECs Exerted Enhanced Proliferation and Increased Telomerase Activity

**Figure 3:**
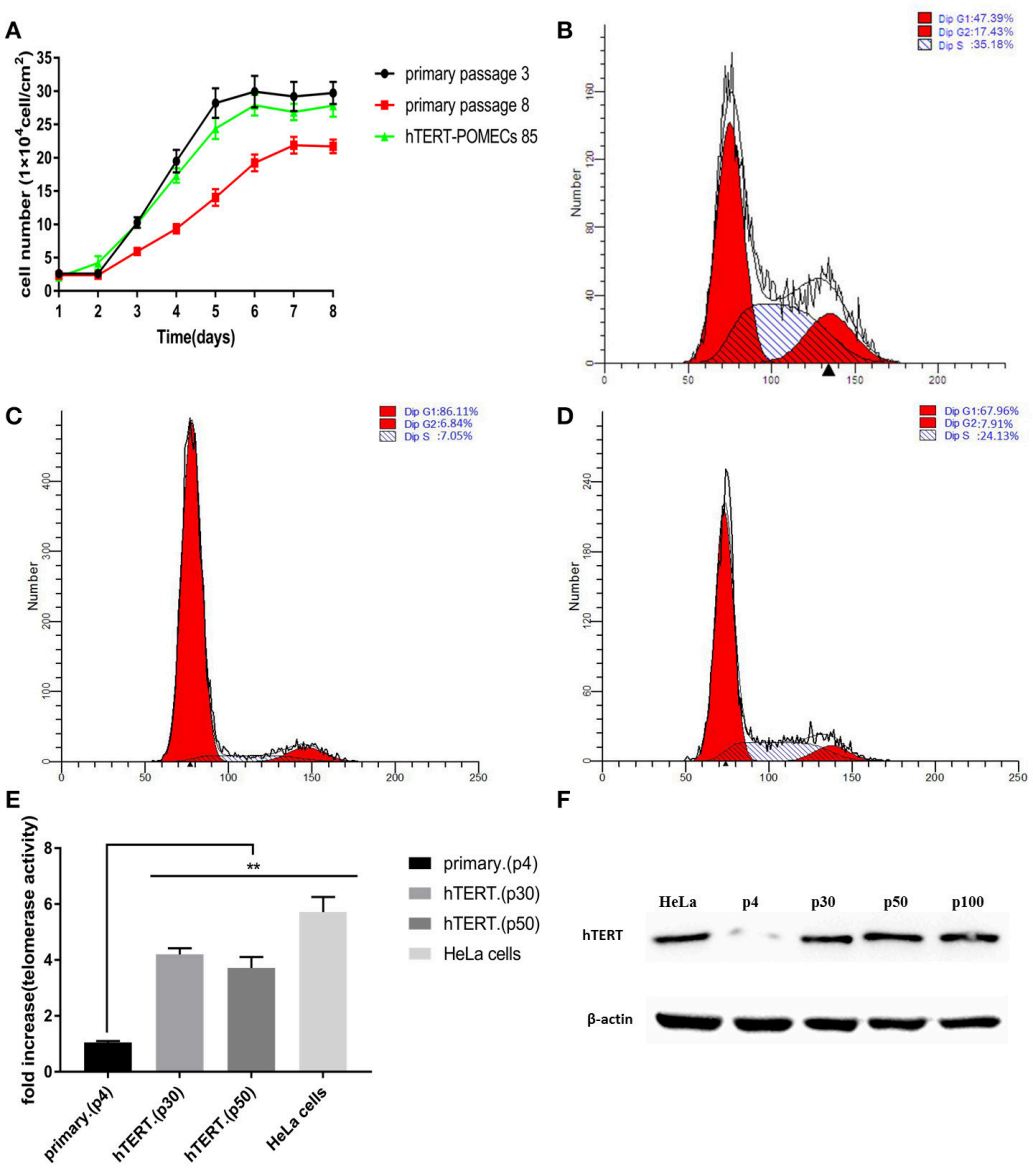
Enhanced proliferation and increased telomerase activity in hTERT-POMECs. **(A)** Growth kinetics of 3rd and 8th passage POMECs and 85th passage hTERT-POMECs. **(B–D)** Cell cycle analysis of 3rd and 8th passage POMECs and 85th passage hTERT-POMECs. The percentages of 3rd and 8th passage POMECs and 85th passage hTERT-POMECs in S phase were 35.18, 7.05, and 24.13%, respectively. **(E)** Telomerase activity in 4th passage POMECs and 30th and 50th passage hTERT-POMECs. HeLa cells served as a positive control. The data were means ± SEM values of three independent experiments. **P < 0.01 vs. fourth passage POMECs. **(F)** Expression of hTERT protein was detected by western blotting. hTERT protein was positively detected in 30th, 50th, and 100th passage hTERT-POMECs, but not in fourth passage POMECs. HeLa cells served as a positive control.

The growth curves of the primary POMECs and hTERT-POMECs are shown in Figure 3A. The eighth passage normal POMECs had an obvious decreased proliferation rate compared with the third passage cells, indicating that the primary POMECs gradually lost their ability to multiply as cell division progressed. Overexpression of hTERT alone can rescue the propagation deficiency. The 85th passage hTERT-POMECs almost had the same proliferation ability as the third passage POMECs (Figure 3A).

To confirm whether hTERT expression affected cell proliferation or cell cycle progression, third and eighth passage normal POMECs and 85th passage hTERT-POMECs were determined by flow cytometry. A significant difference was observed between the cell cycles of eighth passage POMECs and third passage POMECs or 85th passage hTERT-POMECs when they were cultured under the same conditions (Figures 3B–D). The third passage POMECs and 85th passage hTERT-POMECs showed a robust proliferating ability, while the eighth passage POMECs had a growth arrest phonotype. The percentage of cells in S phase in 85th hTERT-POECs (24.13%) was higher than that in eighth passage POMECs (7.05%), suggesting that hTERT-POMECs have overcome the replicative senescence crisis.

Telo TAGGG Telomerase PCR ELISA^PLUS^ kit was employed to evaluate the telomerase activity in hTERT-POMECs. Telomerase activity of the 30th and 50th passage hTERT-POMECs was 4.20-fold and 3.71-fold higher than that in the fourth passage POMECs (*P* < 0.01), respectively (Figure 3E). Western blot analysis further showed that the exogenous hTERT gene was stably expressed in early or late passage hTERT-POMECs, but not in the fourth passage primary POMECs (Figure 3F). In conclusion, these results confirmed increased telomerase activity and enhanced proliferation in hTERT-POMECs.

### Newly Established hTERT-POMECs Are Immortalized but Not Transformed

**Figure 4:**
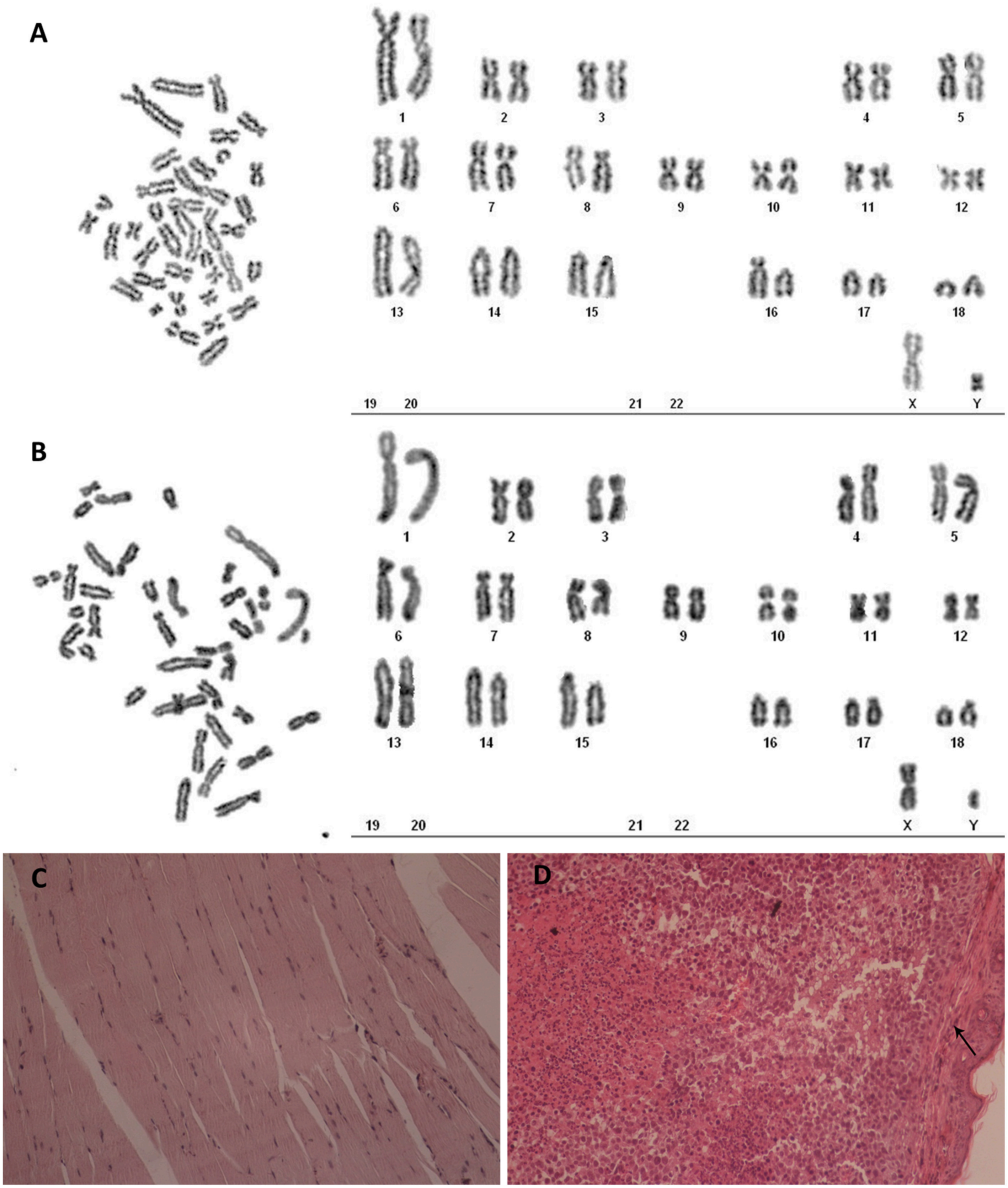
Analysis of karyotype and tumorigenicity in hTERT-POMECs. **(A,B)** Karyotype analysis of third passage POMECs and 100th passage hTERT-POMECs. They both showed normal diploid numbers of chromosomes, 2n = 36+XY pattern. **(C,D)** Tumorigenicity assays. Eightieth passage hTERT-POMECs and HeLa cells were injected subcutaneously into nude mice. One month later, the hTERT-POMECs group did not form tumors, and histological examination revealed a normal tissue structure at the site of hTERT-POMECs injection (**C**, ×100 magnification). However, HeLa cells formed tumors under the skin (the black arrows indicate the skin), which displayed high nuclear/cytoplasmic ratios and irregular dense tumor cell invasion (**D**, ×100 magnification).

Karyotype analysis was carried on third passage POMECs and the 100th passage hTERT-POMECs. Third passage POMECs had a diploid chromosome complement of 2n = 38, consisting of one pair of sex chromosome (X, Y) and 18 pairs of autosomes (Figure 4A). No chromosome abnormality was found in the 100th passage hTERT-POMECs compared to the primary POMECs (Figure 4B). To determine whether the immortalized hTERT-POMECs are transformed, tumorigenicity assays were carried out by injecting either hTERT-POMECs (80th passage) or HeLa cells (positive control) subcutaneously into nude BALB/c mice. Tumors were observed with 14 days in the mice injected with HeLa cells, while no tumors were found in the mice injected with hTERT-POMECs. Further histological examination revealed that a normal tissue structure at the site of hTERT-POMECs injection (Figure 4C), whereas neoplasms with irregular dense tumor cell invasion were observed at the injection site of HeLa cells (Figure 4D). These results indicated that the hTERT-POMECs had no tumorigenic transformation.

### PCV2 Infects hTERT-POMCs and Induces Cytopathogenic Effects

**Figure 5:**
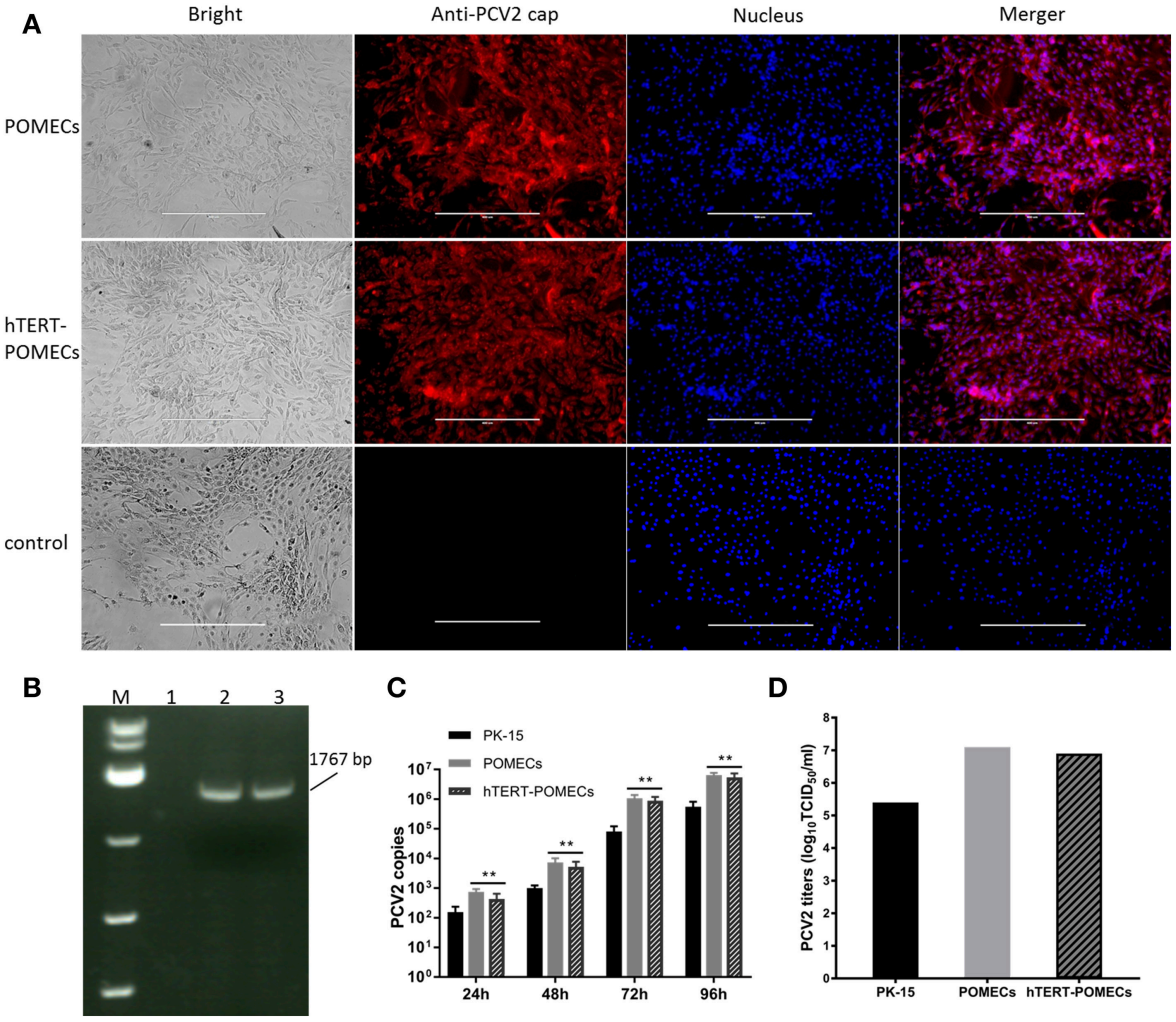
Infection of PCV2. **(A)** Detection of PCV2 by immunofluorescence. Third passage POMECs and 70th passage hTERT-POMECs were infected with PCV2 at a MOI of 1, and anti-PCV2 Cap protein antibodies was used to detect PCV2 at 24 hpi. Uninfected hTERT-POMECs served as negative control (×100 magnification, scale bar is 400 μm). **(B)** Detection of PCV2 by RT-PCR. PCV2 intact genome was positively detected in the infected POMECs (line 2) and hTERT-POMECs (line 3), but not in uninfected hTERT-POMECs (line 1). M: DL 5000(Takara). **(C)** Analysis of PCV2 replication in PK-15, POMECs and hTERT-POMECs at 24, 48, 72, 96 hpi by real-time quantitative PCR. The data shown are representative of three independent experiments. **P < 0.01 vs. PK-15 cells under the same condition. **(D)** Viral titers of PCV2 propagated in PK-15, POMECs and hTERT-POMECs at 96 hpi.

**Figure 6:**
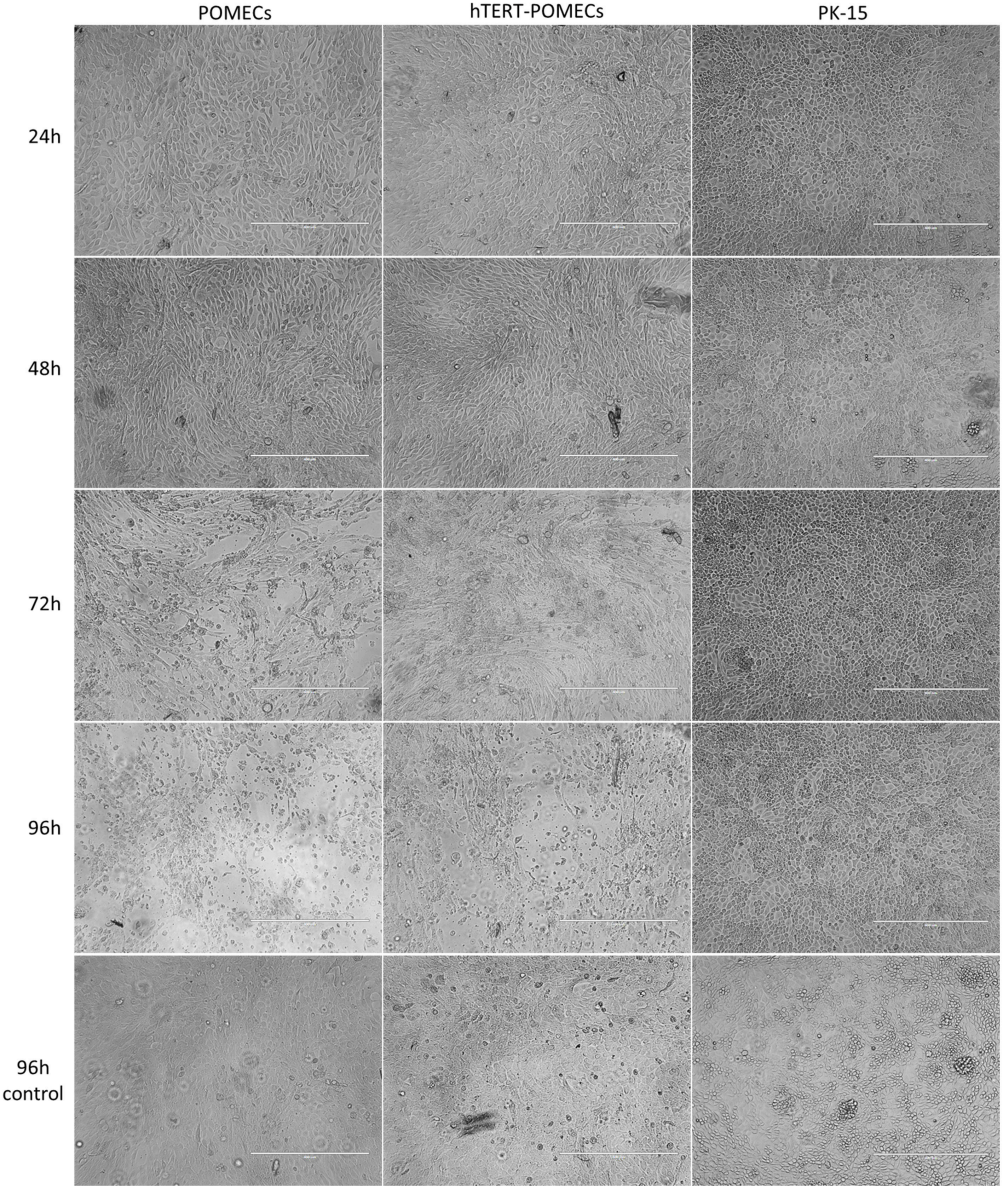
Cytopathogenic effects in PCV2-infected hTERT-POMECs. Third passage POMECs, 70th passage hTERT-POMECs and PK-15 cells were infected with PCV2 at a MOI of 1. Cells were observed at 24, 48, 72, and 96 hpi. At 24 and 48 hpi, the PCV2-infected POMECs and hTERT-POMECs were elongated, and the intercellular space increased. At 72 and 96 hpi, visible cytopathogenic effects were observed in POMECs and hTERT-POMECs. No cytopathogenic effect was found in PK-15 cells. Uninfected POMECs, hTERT-POMECs and PK-15 cells served as controls (×100 magnification, scale bar is 400 μm).

PCV2 is the primary etiological agent of PCV-associated disease in swine. However, knowledge of the mechanism of PCV2 pathogenesis is restrained by the lack of an ideal cell model. Occasionally and repeatedly, we found that POMECs showed obvious CPE with infection of PCV2. We confirmed PCV2 was the CPE causative agent by immunofluoresence and PCR (Figures 5A,B). Since hTERT-POMECs retained the morphological and physiological characteristics of primary POMECs, we speculated that PCV2 could also infect and replicate in hTERT-POMECs. As speculation, PCV2 Cap protein was detected by immunofluorescence staining (Figure 5A) and PCV2 intact genome was determined by reverse transcription (RT)-PCR (Figure 5B) in PCV2-infected hTERT-POMECs, but not in uninfected hTERT-POMECs. Compared among bright field images in Figure 5A, PCV2-infected cells had a slight change in morphology and the intercellular space was larger between PCV2-infected cells than non-infected control cells, which was further confirmed in Figure 6. To analyze the replicative capacity of PCV2 in POMECs, hTERT-POMECs and PK-15 cells, DNA of PCV2-infected cells at 24, 48, 72, 96 hpi was extracted and quantified by real-time quantitative PCR. As shown in Figure 5C, PCV2 propagated with time and had higher numbers of genomic copies in primary POMECs and hTERT-POMECs than in PK-15 cells.

It is commonly believed that PCV2 infection does not lead to cytopathic effects in host cells. Surprisingly, we found that PCV2-infected primary POMECs and hTERT-POMECs showed cytopathic-like effects. To confirm this, third passage POMECs, 70th passage hTERT-POMECs and PK-15 cells were infected with PCV2 at a multiplicity of infection of 1. At 24 hpi, the POMECs and hTERT-POMECs were elongated, and the intercellular space increased. These changes aggravated at 48 hpi. Visible cytopathogenic effects in POMECs and hTERT-POMECs were observed at 72 hpi, but not in control and PK-15 cells (Figure 6). Viral titers of primary POMECs and hTERT-POMECs-propagated PCV2 determined by observing CPE on corresponding cells were 1 × 10^7.1^ 50% tissue culture infective dose (TCID_50)_/ml and 1 × 10^6.9^ TCID_50_/ml, respectively, nearly the same to the titration on PCV-free PK-15 cells by immunofluorescence assay. Compared to PK-15-propagated PCV2, which had a titer of 1 × 10^5.4^ TCID_50_/ml titrated by immunofluorescence assay, PCV2 showed better replication and higher titers in primary POMECs and hTERT-POMECs (Figure 5D). Taken together, we demonstrated that PCV2 could infect and replicate in hTERT-POMECs, resulting in cytopathic effects and infectious progeny virus.

## Discussion

The proliferation of normal cells is strictly controlled, otherwise they become cancerous (Ratsch et al., 2001; Zhao et al., 2015). Normal cells have a limited lifespan, undergoing replicative senescence and death after a certain number of divisions (Munoz-Espin et al., 2013; Birch et al., 2018). Previous studies have confirmed that progressively shortened telomeres may be the main cause of cellular senescence (Harley, 1991; di Fagagna et al., 2003). The telomeres lose 30–200 bp each time that cells divide (Harley, 1991). Eventually, the shortened telomeres cannot protect chromosomes from nuclease degradation, inter-chromosomal fusion, and improper recombination, the cells become senescent (Cukusic et al., 2008; Misri et al., 2008). Telomerase, a ribonucleoprotein complex, can prolong the length of telomeres and prevent cell aging (Counter, 1996; Zhang et al., 2016, 2017). hTERT, a catalytic subunit of the telomerase, can make up for the telomere shortening caused by cell division and extend the lifespan of cells. In this study, pCI-neo-hTERT plasmids were successfully introduced into primary POMECs and immortalized the cells. PCRs and telomerase activity assays indicated that the hTERT gene was stably expressed in hTERT-POMECs. To date, the established immortalized hTERT-POMECs have been cultured for >150 passages without morphological changes. Proliferation assays and flow cytometry demonstrated that hTERT-POMECs had a better multiplication capacity than primary POMECs. Karyotype analysis and tumorigenicity assays indicated that hTERT-POMECs had normal numbers of chromosomes and no tumorigenic transformation.

Unlike vascular, intestinal mucosal, alveolar and other simple columnar epithelia, oral mucosal epithelia are formed of stratified keratinized epithelial cells. In fact, oral mucosal epithelia have more similarities with skin (Squier and Kremer, 2001; Arda et al., 2014), and are composed of basal, spinous, granular and cuticle layers from the inside out. Only the basal layer epithelia have the ability to proliferate and differentiate. Therefore, isolation and cultivation of basal cells is the critical step in primary culture of OMECs.

A common and unavoidable problem in primary epithelial cell culture is fibroblast contamination, which is usually treated by scraping or differential citrate digestion. Dispase II is a neutral protease that hydrolyzes the N-terminal peptide bonds of non-polar amino acid residues. It is gentle and does not damage cell membranes, so dispase II is a good tool to separate the epidermis from the adjacent connective tissue. In the present study, we treated the buccal mucosal tissues with 0.25% dispase II solution and then used tissue culture, avoiding fibroblast contamination from the beginning.

Generally, the basal proliferative layer of OMECs strongly express the keratin pair K5/K14, while the suprabasal differentiated cells strongly express K4/K13 (Hosaka et al., 1985; Belaldavar et al., 2016). In the present study, hTERT-POMECs, as well as the primary POMECs, expressed K5/K14 and K4/K13, as described previously (Mackenzie et al., 1991). The primary POMECs and hTERT-POMECs stably expressed K14 and K13, indicating that they both had the characteristics of basal and suprabasal cells, retaining the potential for proliferation and differentiation. Karyotype analysis showed that there were no chromosomal changes in hTERT-POMECs, and tumorigenicity assays indicated that the cells were not neoplastically transformed. Therefore, the immortalized cell line hTERT-POMEC may provide a good cell model for research on pathogenesis in swine.

PCV2 is the major infectious pathogen of PCVADs, which cause major economic loses in the swine industry worldwide (Gillespie et al., 2009; Ge et al., 2012). Lack of a consistent cytopathogenic cell model has seriously restricted the study of PCV2 pathogenesis. To date, no cell models have been found to be cytopathogenic following PCV2 infection. In this study, we surprisingly discovered that PCV2 propagated in the immortalized hTERT-POMECs as well as primary POMECs. Real-time quantitative PCR and TCID_50_ assay demonstrated that PCV2 had a higher replication level in POMECs and hTERT-POMECs than in PK-15 cells. Importantly, we observed cytopathogenic effects in primary POMECs and hTERT-POMECs, which can provide an ideal cell model for studying the pathogenesis of PCV2.

In conclusion, we established an immortalized POMEC line, hTERT-POMEC, by transfecting the eukaryotic expression plasmid pCI-neo-hTERT into primary POMECs. hTERT-POMECs retained the morphological and physiological characteristics of primary POMECs, but had better propagation performance without transformation. Virus infection assays showed that PCV2 replicated well in hTERT-POMECs, and obvious cytopathogenic effects were observed in hTERT-POMECs as well as primary POMECs. Taken together, all the results indicate that the hTERT-POMEC line is a suitable cell model to investigate physiological and pathophysiological processes of PCV2 infection.

## Materials and Methods

### Ethics Statement

Neonatal, unsuckled piglets were obtained from the Laboratory Animal Center of Northwest A&F University, China. Athymic nude BALB/c mice, aged 4 weeks, were purchased from the Animal Center of the Fourth Military Medical University (Xi'an, China). This study was carried out in accordance with the recommendations of Guidelines for the Care and Use of Animals of Northwest A&F University. The protocol was approved by the Animal Care and Use Committee of Northwest A&F University, China.

### Isolation and Cultivation of Primary POMECs

Buccal mucosal tissues were collected from a neonatal, unsuckled piglet. The tissues were rinsed five times by phosphate buffer solution (PBS) and cut into 0.2 × 1-cm segments, and then placed into 0.25% dispase II (Sigma–Aldrich, St. Louis, MO, USA) solution at 4°C. After 20 h gentle digestion, the upper epidermal cells were torn off from the connective tissue below. The epidermis was gently removed with tweezers and cut into small fragments (volume <1 mm^3^). Epidermal fragments were washed twice in PBS, once in Dulbecco's modified Eagle's medium/Nutrient Mixture F-12 (DMEM/F12), and resuspended in DMEM/F12 containing 2 mM l-glutamine, 100 U/ml penicillin, 100 μg/ml streptomycin, 20 ng/ml epidermal growth factor, ITS (insulin 1.0 g/L, transferrin 0.55 g/L and selenium 0.67 mg/L), and 10% heat-inactivated fetal bovine serum, and incubated at 37 °C in a humidified incubator with 5% CO_2_. The medium was replenished every 3 days. Cells at 70%−80% confluence were digested with 0.25% trypsin and subcultured in a ratio of 1:3.

### Cell Transfection and Selection

The third generation of POMECs was plated in 12-well plates at 10^4^ cells/cm^2^ and cultured in DMEM/F12 medium without antibiotics. The pCI-neo-hTERT plasmids were transfected into POMECs using Lipofectamine 2000 (Invitrogen, Carlsbad, CA, USA) when the cells reached 90% confluence. Four hours following transfection, the medium was changed to fresh DMEM/F12. After 48 h incubation, the transfected cells were selected with DMEM/F12 containing 500 μg/ml G418 (Sigma–Aldrich). The selective medium was replenished every other day. After 14 days, the surviving cells were maintained in 250 μg/ml G418 to ensure stable selective conditions, and the positive cells were propagated in further culture.

### Immunofluorescence

Cells cultured in 60-mm dishes were fixed with stationary liquid (methanol and acetone, v/v = 1:1) for 20 min at −20°C. The cells were treated with 1% Triton X-100 in PBS for 10 min at room temperature. After blocking with 5% skimmed milk, cells were incubated with the primary antibodies at 4°C overnight. The following primary antibodies were used at a dilution of 1:500: anti-pan-cytokeratin, anti-cytokeratin 13, anti-cytokeratin 14, anti-vimentin, anti-cytokeratin 18 (All obtained from Abcam, Cambridge, MA, USA), and anti-PCV2b Cap antibody (kindly provided by Dr. Shuanghui Yin, Lanzhou Veterinary Research Institute, China). Protein staining was detected using secondary fluorescein isothiocyanate (FITC)-conjugated goat anti-mouse IgG or FITC-conjugated goat anti-rabbit IgG (1:100, Tianjin Sungene Biotech, China) for 1 h. The cells were observed and imaged by Evos f1 fluorescence microscope (Advanced Microscopy Group, Mill Creek, WA).

### Cell Proliferation Assay

Cells were seeded in 24-well plates at 2 × 10^4^ cells/ml and counted daily for 8 days. Three independent experiments were performed in triplicate. The growth kinetics of the cells were plotted.

### Flow Cytometry

To determine the cell cycle of newly established immortalized hTERT-POMECs, flow cytometry was performed. Cells in good condition were collected, fixed with 70% ethanol, and then stained by propidium iodide (Sigma–Aldrich). Cell cycles were measured using a Coulter Epics XL FACS (Beckman Coulter, Brea, CA, USA).

### Telomerase Activity Assay

Telomerase activity of primary POMECs and hTERT-POMECs was analyzed using the Telo TAGGG Telomerase PCR ELISA^PLUS^ kit (Roche, Mannheim, Germany) according to the manufacturer's instructions. Telomerase-positive HeLa cells were used as a positive control and a negative control with enzyme-inactivated sample material was performed according to the instructions. Briefly, the extract of equivalent cells was harvested for polymerase chain reaction (PCR) amplification. Then the PCR products were denatured and hybridized to digoxigenin-labeled detection probes for telomeric repeat-specific ELISA. The absorbance of samples was measured with a microplate spectrophotometer (Infinite 200 PRO Nano-Quant, Tecan, Switzerland) at 450 and 690 nm.

### Western Blotting

Expression of hTERT in immortalized POMECs was examined by western blotting. Cells were digested by trypsin and rinsed twice with PBS. RIPA buffer containing 1 mM phenylmethylsulfonyl fluoride (Solarbio, Beijing, China) was used for cellular protein extraction. Equivalent amount of proteins samples was separated by 10% SDS PAGE and transferred to a polyvinylidene fluoride membrane. After washing and blocking, membranes were incubated with anti-β-actin (1:500, Santa Cruz Biotechnology, Santa Cruz, CA, USA) or anti-telomerase reverse transcriptase antibody (1:1,000, Abcam) at 4°C overnight. Blots were detected with Western Light kit (Advansta, Menlo Park, USA) after membranes were incubated with secondary antibodies (horseradish-peroxidase-labeled goat anti-mouse or rabbit IgG [1:5,000, Bioss, Beijing, China)] for 1 h at RT.

### Karyotype Analysis

The chromosomes of primary POMECs and hTERT-POMECs were determined by karyotype analysis as previously described (Hong et al., 2007). Briefly, cell cultures of third passage POMECs and 100th passage hTERT-POMECs in the exponential phase of growth were treated with colchicine (0.01 μg/ml) at 37°C for 6 h. After trypsinization and collection, the cells were incubated in hypotonic salt solution (0.04 M potassium chloride) at 37°C for 20 min and fixed in ice-cold acetic acid/methanol (v/v = 1:3) for 20 min. Then the fixed cells were collected by centrifugation and suspended in 1 mL PBS. The dispersed cell suspension was dropped on a cold slide, air dried and stained with Giemsa. Metaphase chromosomes were observed and karyotypes were analyzed using ASI Chromosome Analysis System (Israel).

### Tumorigenicity Assay

Tumorigenicity refers to the ability of cultured cells to develop viable tumors in immune-deficient animals. To analyze the potential tumorigenicity of hTERT-POMECs, 1 × 10^6^ cells at 80th passage were injected subcutaneously into the flanks of three nude mice (4 weeks old). Equivalent HeLa cells were injected into three other nude mice as positive controls. All the mice were monitored for 1 month to examine tumor formation and sacrificed for further histological examination.

### PCV2 Infection Assay and Titration

PCV2 virus (GenBank accession number FJ948167) was kindly provided by Dr. Shuanghui Yin. PCV2 was propagated in PK-15 cells, the infected cells and supernatants were harvested at 72 hpi. PCV-free PK-15 cells were used to determine the titer of the obtained PCV2 by immunofluorescence assay because PCV2 could not cause CPE on PK-15 or other experimental cells currently. Since primary POMECs and hTERT-POMECs infected with PCV2 showed obvious CPE, primary POMECs and hTERT-POMECs-propagated PCV2 was titrated by observing CPE on corresponding cells. DNA of PCV2-infected cells (PK-15, POMECs, hTERT-POMECs) at 24, 48, 72, 96 hpi was extracted by the TaKaRa DNA Mini kit (Takara, Dalian, China). Quantification of PCV2 DNA was assayed by real-time PCR with the TaKaRa SYBR Green qPCR Kit (Takara). The purified DNA was treated with RNaseH (Sigma–Aldrich) at 37 °C for 20 min to eliminate RNA interference. A 117 bp region from ORF2 gene of PCV2 was amplified with a primer pair (forward: TAGTATTCAAAGGGCACAG; reverse: CAAGCGAACCACAGTCAA). A recombinant pMD19 plasmid vector (Takara) containing PCV2 genome inserts was used as the standard. Other primers used for RT-PCR are listed in Table 1.

**Table 1:** Primers used for PCR in this study.

**Primers**	**Sequence (5^′^ → 3^′^)**	**Purpose**
TLR 1-F	GTGTTGCCAATCGCTCAT	Detection of TLR1
TLR 1-R	CCAGATTTACTGCGGTGC	
TLR 2-F	GGAGCCTTAGAAGTAGAGTTTG	Detection of TLR2
TLR 2-R	TGTATCCACATTACCGAGGG	
TLR 3-F	TAACAACCTTCCAGGCATA	Detection of TLR3
TLR 3-R	AAGAGGAGAATCAGCGAGTG	
TLR 4-F	TCTACATCAAGTGCCCCTAC	Detection of TLR4
TLR 4-R	ATTCTCCCAAAACCAAC	
TLR 7-F	CATGTGATCGTGGACTG	Detection of TLR7
TLR 7-R	GTGATGCTCGCTATGTGG	
RIG-I-F	CCATTGAAAGTTGGGATT	Detection of RIG-I
RIG-I-R	TGGGCTGTAAGTATGAGGT	
MDA 5-F	TGGGAACATAAACGC	Detection of MDA 5
MDA 5-R	GTCAACAGTTGCTCCTC	
IL-1β-F	CAGCACCTCTCAAGCAGAACAA	Detection of IL-1β
IL-1β-R	GGCAGCAACCATGTACCAACT	
IL-6-F	GAGCCCACCAGGAACGAAAGAG	Detection of IL-6
IL-6-R	GCAGTAGCCATCACCAGAAGCA	
IFN-β-F	CATCCTCCAAATCGCTCTCC	Detection of IFN-β
IFN-β-R	CTGACATGCCAAATTGCTGC	
TNF-α-F	CACCACGCTCTTCTGC	Detection of TNF-α
TNF-α-R	CCTCGGCTTTGACATT	
Defensin-β3-F	AAGTCTACAGAAGCCAAAT	Detection of defensin-β3
Defensin-β3-R	GGTAACAAATAGCACCATAA	
PCV2-F	CTTTTTTATCACTTCGTAATGGTTT	Detection of PCV2
PCV2-R	ACTCAGTAATTTATTTCATATGGAAATT	

### Statistical Analysis

Data from the cell proliferation assay, telomerase activity assay, and real-time PCR were analyzed by SPSS software. All data are presented as mean ± SEM. Differences between groups were compared by one-way analysis of variance. *P* < 0.05 was considered to be statistically significant.

## Data Availability

The raw data supporting the conclusions of this manuscript will be made available by the authors, without undue reservation, to any qualified researcher.

## Author Contributions

HC, KG, and YZ designed the study. HC performed the experiments with the help from WL, DW, KG, and HC wrote the final manuscript. KG and YZ revised the manuscript.

### Conflict of Interest Statement

The authors declare that the research was conducted in the absence of any commercial or financial relationships that could be construed as a potential conflict of interest.
